# Current state of the use of antibiotic prophylactics in cardiac surgery – correspondence

**DOI:** 10.1097/JS9.0000000000000445

**Published:** 2023-05-09

**Authors:** Toufik Abdul-Rahman, Murad O. Omran, Olabode Ekerin, Shankhaneel Ghosh, Wireko A. Awuah

**Affiliations:** a Medical Institute, Sumy State University, Sumy, Ukraine; b Department of Physiology, Faculty of Medicine, University of Malaya, Kuala Lumpur, Malaysia; c College of Medicine, Lagos State University, Lagos, Nigeria; d Institute of Medical Sciences and SUM Hospital, Siksha ‘O’ Anusandhan, Bhubaneswar, India

*Dear Editor,*

Antibiotic prophylaxis is critical in cardiac surgery to prevent surgical site infections (SSIs) and improve patient outcomes. However, the overuse of antibiotics can lead to antibiotic resistance, making it challenging to treat infections in the future. This editorial aims to provide an overview of the current state of antibiotic prophylaxis in cardiac surgery, including the types of antibiotics used and issues associated with their use.

SSIs are one of the most prevalent infections in healthcare settings, intricately linked to poor prognosis. It generally affects about 10–20% of patients who undergo surgery, with incidence rates that fluctuate between 3.5 and 26.8% exclusively for those who undergo cardiac surgeries^1^. The most common forms of SSI in cardiac surgery patients include graft harvesting sites and sternal wound infections. To prevent SSI, several measures are employed preoperatively, intraoperatively, and postoperatively in cardiac surgery including antibiotic prophylaxis^1^. Cephalosporins, vancomycin, and aminoglycosides are examples of antibiotics currently used for SSI prophylaxis in cardiac surgery. However, the effectiveness of these antibiotics varies, and some bacteria are becoming resistant to them. A recent study by de Tymowski *et al*. investigated the impact of antibiotic prophylaxis on SSIs in cardiac surgery. The study concluded that antibiotic prophylaxis is associated with a lower incidence of SSIs and reduced postoperative complications. However, the study also highlighted the need for proper antibiotic stewardship and the risk of antibiotic resistance^2^.

Antibiotic resistance is currently a growing concern in cardiac surgery and SSI management. The increasing use of antibiotics has led to the emergence of antibiotic-resistant bacteria, such as methicillin-resistant *Staphylococcus aureus*. These bacteria can cause severe infections that are difficult to treat, leading to prolonged hospital stays, higher healthcare costs, and increased mortality rates. According to Zukowska and Zukowski^1^, the emergence of antibiotic-resistant bacteria has decreased the effectiveness of antibiotics used in cardiac surgery. Therefore, the judicious use of antibiotics is essential to prevent the development of antibiotic-resistant strains. The impact of other factors that can influence the effectiveness of prophylactic antibiotics on SSI rates, such as dosing, duration, and adherence to guidelines, have been well established in the literature. A systematic review by Amato *et al*.^3^ evaluated perioperative antibiotic prophylaxis in vascular surgery and found that adherence to guidelines and duration of prophylaxis may impact the incidence of groin SSI. Moreover, a study by Hamouda *et al*. compared different perioperative antibiotic prophylaxis duration strategies in adult patients undergoing cardiac surgery. The study concluded that a shorter duration of antibiotic prophylaxis was associated with similar outcomes compared to a longer duration^4^. Therefore, practitioners should consider administering shorter antibiotic prophylaxis in cardiac surgery while ensuring appropriate antibiotic stewardship measures are taken. Another study on perioperative antibiotic prophylaxis recommendations in various surgical specialities found that adherence to guidelines is critical in preventing postoperative SSI^5^.

Given the increasing concern over antibiotic resistance, there is a need for continued research on the optimal use of antibiotics in cardiac surgery. This includes investigating alternative antibiotic regimens, such as the use of narrow-spectrum antibiotics or combination therapy, as well as the use of non-antibiotic strategies, such as probiotics, to prevent SSIs. In addition, efforts to improve antibiotic stewardship and reduce unnecessary antibiotic use in cardiac surgery should be a priority.

## Ethical approval

Not applicable.

## Consent

Not applicable.

## Sources of funding

The authors received no external funding for this project.

## Author contribution

T.A.-R.: conceptualization of ideas; T.A.-R.: data curation; M.O.O., O.E., and S.G.: writing of initial draft; T.A.-R. and W.A.A.: review and editing.

## Conflicts of interest disclosure

The authors declare no conflicts of interest.

## Research registration unique identifying number (UIN)

Not applicable.

## Guarantor

Toufik Abdul-Rahman.

## Data availability statement

The authors declare that no new data were generated.

## Provenance and peer review

The paper was not invited.

## Acknowledgements

The authors would like to acknowledge Toufik’s World Medical Association and ICORMed Collaborative for organizing this project.
